# The beauty of the beast: Suggestions to curb the excesses of dog breeding and restore animal welfare – Invited review

**DOI:** 10.17221/62/2024-VETMED

**Published:** 2024-11-28

**Authors:** Claire Diederich

**Affiliations:** Department of Veterinary Medicine (NARILIS-IVRU), Faculty of Sciences, University of Namur, Namur, Belgium

**Keywords:** canine welfare, dog-human relationship, genetic defect, selective breeding

## Abstract

*Dog. Specifically created to save its master’s life. – (The dog is the ideal) Friend of man, (because it is his devoted slave)* (source: Gustave Flaubert, Dictionnaire des Idées Reçues). But is man the best friend of the dog? This question is legitimate when we consider living situations to which modern domestic dogs are exposed. They often do not satisfy basic animal needs. In this narrative review, the author revisits the history of the dog’s presence alongside humans, in the light of current knowledge. The modern dog (breed standards and their interests in canine research) and its breeding strategy, including extreme breeding, will then be given particular attention. Dysfunctional human psychological processes will be explored to make it possible to grasp why the breeding of the modern dog is undergoing such a transformation. Finally, based on these factual and conceptual insights, suggestions to improve canine welfare will be proposed. To be effective, all these must be assessed against real-world conditions.

## INTRODUCTION

*Dog. Specifically created to save its master’s life. – (The dog is the ideal) Friend of man, (because it is his devoted slave)* (source: Gustave Flaubert, Dictionnaire des Idées Reçues). But is man the best friend of the dog? This question is legitimate when we consider the situations to which modern domestic dogs are exposed, often without fulfilling their basic ethological needs. Indeed, the living environment of companion dogs varies greatly in both quantity and quality: from rural to urban areas, access to the outdoors (free access or not, on or off leash), presence of other animals (conspecifics and/or others), and humans (the owner and other people). Some owners are concerned with stimulating their animal’s cognitive abilities, engaging in shared activities, house training as well as more specialised training (e.g., Agility, Dog Dancing, Fly-Ball, protection, hunting, and various services). While a link exists between the owners’ emotional state and their dog’s behavioural or health problems (Barcelos et al. 2023); others do not hesitate to abandon them in shelters due to behavioural incompatibilities (Patronek et al. 2022).

Based on the aforementioned points, it can be observed that dogs exhibit remarkable behavioural plasticity (Ophorst and Bovenkerk 2021). While the vast majority of their recent ancestors were engaged as co-workers alongside humans, helping to reduce their daily workload and earning their daily sustenance, this is no longer the case today. Due to society’s critical view on how humans currently treat animals, the welfare of working dogs has received the attention it deserves (Cobb et al. 2021). However, this is not true for the rest of the canine population living in our industrialised societies, whose primary function is to keep humans company (when the latter are present). Nevertheless, while the optimal canine has been delineated by King et al. (2009) (i.e., medium-sized, low-maintenance (short hair), neutered, sociable, obedient, clean, safe with children, and in good health), the optimal environment for a pet dog remains undefined. The living environments of animals and their relationships with their owners are highly diverse, which may be a contributing factor to this diversity. In a study conducted by Westgarth et al. (2019), interviews with pet owners revealed that the perception and interpretation of the concept of “responsible ownership” varies considerably depending on the individual in question. The authors identified four elements that qualify this concept: the relationship between the owner and the dog (whether it is too strong or too weak), the owner’s perception of what is good or bad for the dog, the owner’s ability to avoid or prevent conflicts with the dog, and the owner’s degree of tolerance regarding the inconveniences associated with the dog’s presence. Theoretically, all dog breeds are adaptable to companionship. Ghirlanda et al. (2013), in their study of the popularity of dog breeds (using four popularity indices calculated from the number of registered dogs per breed in the American Kennel Club data bank, from 1926 to 2005), found that behavioural traits, longevity, and good health are not criteria that favour the popularity of a breed. Surprisingly, the presence of behavioural problems or hereditary diseases does not prevent a breed from becoming popular. In other words, form matters more than substance.

In this narrative review, the author revisits the history of the dog’s presence alongside humans, in the light of current knowledge. The modern dog (breed standards and their interests in canine research) and its breeding – whose strategy, including extreme breeding, will then be given particular attention. Dysfunctional human psychological processes will be explored to make it possible to grasp why the breeding of the modern dog is undergoing such a transformation. Finally, based on these factual and conceptual insights, suggestions to improve canine welfare will be proposed.

## PRESENCE OF THE DOG: HISTORY OF ITS DOMESTICATION

The process of domestication has long been described as the result of the unilateral influence of humans on non-human animals, progressively removing them from their so-called natural (or wild) habitats. Since the last century, the industrialisation of animal production has led to housing animals in modern, fully controlled environments that are often not respectful of their ethological needs. In addition to providing them with shelter and food, as well as protection from predators, disease prevention, and care, humans also control the reproduction of these animals, selecting partners likely to offer the greatest benefits (Purugganan 2022). According to Demoule (2019), the contributions of animals have been classified into those derived from dead animals (e.g. their meat), from living animals (e.g. their milk), from their behavioural skills (e.g. tracking game), or because they hold particular significance (e.g. as offerings to the gods).

This view of an oppressive human dominance over animals, subjecting them to a life of constraints, has been criticised by authors advocating for their fair treatment (e.g. Donaldson and Kymlicka 2011). They question the limits and consequences of these constraints, and the moral nature of human actions on the living world [for an essay review of five recent books on the moral dimensions of human-animal interactions, see Peterson (2021)]. Among these critics, animal rights theorists reject the idea of fair management of domesticated animals by humans. Instead, they advocate for the complete cessation of all domestication, leading to the extinction of domesticated species. This would eliminate the need to concern ourselves with their welfare (Francione 2008).

In recent decades, following research on cognition, emotions, and other aspects of social behaviour in animals, the process of domestication has been enriched by considering them as active participants and influencers in this process, a concept known as animal agency. According to Purugganan (2022), domestication is thus understood as “*a coevolutionary process that arises from a mutualism, in which one species (the domesticator) constructs an environment where it actively manages both the survival and reproduction of another species (the domesticate) in order to provide the former with resources and/or services*” (p. 664).

Thus, a species may have allowed itself to be domesticated because it was willing, deriving certain benefits from the process and influencing the human as the domesticator (Meijer and Bovenkerk 2021). Based on this, it can be hypothesised that thousands of years ago, the wolf (*Canis lupus lupus*) was the most receptive canid to domestication, becoming the sole ancestor of the dog (*Canis lupus familiaris*) (Tancredi and Cardinali 2023).

The process of canine domestication remains hypothetical but may have occurred in two phases, involving the co-evolution of humans and animals over several thousand years. The first phase saw wolves living near humans, who led a roaming hunter-gatherer lifestyle. The second phase involved humans selecting certain wolves with desirable physical or behavioural traits during their transition to a sedentary, agricultural society (Dobney and Larson 2006; Range and Marshall-Pescini 2022). This transition likely moved from wolves to a proto-dog and eventually to the domesticated dog.

For example, these early domesticated dogs were useful to humans for hunting, protection, vermin control, and companionship in various parts of the world, though their precise origin is still debated [in Europe, the High Arctic, and/or East Asia, according to Frantz et al. (2016); Freedman and Wayne (2017); Botigue et al. (2017); Ni Leathlobhair et al. (2018)]. They likely resembled current feral dogs observed in India, South America, or certain islands [e.g., the Antilles, and the Pacific Islands (Botigue et al. 2017)].

Gradually, canine morphotypes as we know them today began to emerge, evidenced by representations of sighthound-type dogs in the artistic remains of Ancient Egypt and the hunting poem Cynegeticon, with its descriptions of hunting dogs by the Latin poet Grattius Faliscus in the early Common Era (Parker 2012). According to Vonholdt and Driscoll (2016) and Delort (2023), there is no evidence in the following centuries that canine selection took place in any way other than to maintain the working qualities of the parents in their offspring. No particular attention was paid to the animals’ external appearance, as long as one had access to a good hunting or guard dog – ideally the best, the most robust, and/or the most aggressive.

The first English dog show took place in 1843, featuring the Spaniel breed (Sampson and Binns 2007), and the first global canine exhibition occurred in Belgium in 1847, featuring the Pointer breed. During the latter half of the 19^th^ century, the first breed clubs were established, along with their associated breed standards (Parker 2012).

## THE MODERN DOG – BREED STANDARDS & CURRENT INTERESTS IN CANINE RESEARCH

There are several major associations around the world that advocate for modern cynology (i.e., canine science: breeding, education, and training) and dog breeds. These include the Fédération Cynologique Internationale (FCI, which declares itself the World Canine Organisation), the English Kennel Club (KC, the world’s first canine society), the American Kennel Club (AKC), and the United Kennel Club (UKC, the self-declared world’s largest all-breed registry today). Established between the latter half of the 19^th^ century and the early 20^th^ century (Sampson and Binns 2007), these associations recognise respectively 356 (as of 2024, fci.be/nomenclature/), 222 (as of 2022), 200 (as of 2024, akc.org/dog-breeds/), and 308 (as of 2024, ukcdogs.com/breed-standards) dog breeds.

To be classified as a specific dog breed, a dog must meet morphological (primarily) and behavioural (secondarily) criteria that distinguish it from other dogs. These criteria are described in what is called a breed standard. Some criteria are clearly specified (objective criteria), while others are left to the judge’s discretion (subjective criteria). For instance, the desirable adult height at the withers for the Belgian Shepherd is 62 cm for males and 58 cm for females (with a tolerance of minus 2 cm and plus 4 cm) (FCI-St. No. 15/22. 06. 2001). Meanwhile, the judge assesses the topline of the German Shepherd as follows: “*The topline runs without visible interruption from the neck set through the high and long withers and the straight back to the slightly sloping croup. The medium-length back is firm, strong, and well-muscled. The loin is broad, short, strongly developed, and well-muscled. The long and slightly inclined croup (approximately 23° to the horizontal) blends seamlessly into the tail set*“ (excerpt from FCI-St. No. 166/16. 10. 2018). Breed standards are maintained by the country of origin of the respective breed.

When they were first established, dog shows in England provided an opportunity to compete animals both in the show ring and in the field. Hence, dogs were judged on their conformation as well as their performance in field trials, as most were entered in both types of competitions (Sampson and Binns 2007). Today, the range of competitions based on dogs’ skills has expanded. The FCI now organises contests in Beauty, Exhibition, Work, Greyhound Racing, Obedience, Agility, Herding, and Dog Dancing (fci.be/en/FCI-International-Championship). In its recent report on the proportion of pedigree dogs in Europe, the FCI estimates that, on average, 15.36% (S.D. 14.83%) of the canine population consists of pedigree dogs (Marton 2024). Despite this low proportion among the canine population, each breed constitutes a unique set of genes, grouped into a genetic niche. The phenotypic expression of each of these gene groups allows for the unmistakable recognition of the breed.

In recent decades, research on canine genetics has taken multiple directions. It has identified the genes responsible for the phenotypic diversity within hundreds of dog breeds. It has been discovered that, in dogs (unlike in other human and non-human mammals), only a few genes are required to ensure the observed diversity (Plassais et al. 2019). For instance, three genes and three mutations of these genes and their combinations are sufficient to explain the seven varieties of canine coat (Parker et al. 2010). In dogs with genetic diseases, identifying the responsible gene has made it possible to select healthy breeding stock [e.g., primary glaucoma (Komaromy and Petersen-Jones 2015)] or dogs with specific behavioural capacities (e.g., Matsumoto et al. 2023). In line with this, the new knowledge of canine genetics also helped at deciphering metabolic problems with very low prevalence, such as the copper toxicosis and hepatopathies (copper accumulation in the liver) known in several terrier breeds and also in Dalmatians, Labrador Retrievers, and other breeds (see e.g. Corbee and Penning 2021). This problem has been approached by changing canine dry foods (e.g. Fieten et al. 2014) but all veterinarians should be aware of it and advise breeders and their clients buying dogs on how to avoid/limit these genetically based diseases. Indeed, chronic hepatic disease is a serious welfare problem.

Furthermore, dogs can be genetic models for human diseases. The canine genetic target is first identified by comparing the genome of healthy dogs with those suffering from the same disease within the same breed (e.g. Corbee and Penning 2021). It is then possible, through comparative genetics, to search for the implicated gene in humans (Doughman 2019; Kilk 2019; Kaur et al. 2023) and determine whether it is also responsible for a particular (rare) disease e.g. human ichthyoses (Grall et al. 2012) or to better understand certain neuropsychiatric disorders (Morrill et al. 2023).

## BREEDING STRATEGY (AND EXTREME BREEDING)

When humans undertake the selection of animals from a limited population that exhibit traits deemed desirable, they reduce the original genetic variability by increasing inbreeding (Packer 2018). Breeding such selected individuals together allows for the fixation of these traits of interest, while aiming to improve the quality of breeding and ensure its sustainability (Sampson and Binns 2007). In dogs, as far as the author can ascertain, aside from avoiding inbreeding and the transmission of genetic defects (Bannasch et al. 2021), the breeding strategies employed by modern breeders, whether consciously or unconsciously, have not yet been thoroughly researched. Based on methods for eliciting breeding objectives in livestock breeding (Burns et al. 2022), the author suggests that the desirable traits for dog breeders might include these ones: the presence of a phenotypic characteristic that makes the animal unique (e.g., eye colour, coat variety, aptitude for work or sports); well-defined physical traits specific to the breed (e.g., short nose, long back); balanced temperament (e.g., low fear and aggression towards humans); good learning abilities; robust disease resistance; and favourable reproductive qualities (e.g., fertility, fecundity, prolificacy, ease of birthing, maternal behaviour). This suggested list of traits should be validated by additional research. Factors related to the (future) owners of the animals, trends, animal welfare, and quality of life should also be considered. The prioritization of these criteria could be conducted based on their importance to all human stakeholders: breeders, owners, veterinarians, shelters, and various associations.

The selection of partners for the purpose of propagating a breed is a complex task for breeders [for an analysis of this complexity in livestock breeding, see Martin-Collado et al. (2018)]. Aware of the ethical questions raised by the practice of dog breeding and trade (Menor-Campos 2024), canine associations have provided their members with digital tools to assist in the selection of breeding stock, e.g., Mate Select by the Kennel Club (KC), the use of which was analysed by Janes et al. (2020) and is available at thekennelclub.org.uk/dog-breeding/, or in France, LOF Select (centrale-canine.fr/lofselect). In Australia, researchers have adapted a genetic relationship visualization tool originally developed for livestock (NetView) to a subpopulation of German Shepherds (Mortlock et al. 2015). However, problematic situations related to genetic transmission and variability have been revealed. For instance, a high-quality dog (awarded in canine competitions for physical or specific abilities), preferably male due to his ability to produce more offspring than a female, is often selected more frequently, risking the spread of genetic defects it may carry [due to the breeder’s greed or ignorance (Leroy 2011)].

The genetic variability of the canine genome is fragmented among breeds. Globally, individuals of the same breed form genetic subpopulations distributed geographically according to their hosting continent. Each subpopulation evolves independently to varying degrees, depending on the exchanges occurring between subpopulations (Neff et al. 2004; Quignon et al. 2007; Bach 2019). High demand for a particular breed characteristic drives breeders to intensive production, risking inbreeding and the concentration of genetic defects (Axelsson et al. 2021). This demand can also be detrimental to the animal’s welfare when suffering is associated with this specific characteristic. For instance, Bannasch et al. (2021) demonstrated that among 227 dog breeds, each represented by at least 30 individuals, morbidity was positively influenced by both body size and inbreeding, with healthier dogs belonging to smaller and less inbred breeds.

There are hundreds of problems related to a defective genome in dogs. For a targeted search, see specialised websites such as genodog.fr or omia.org. It should be noted that the same hereditary disease can be found in several breeds, and that several hereditary diseases can be expressed within the same breed. Examples of this include, in some breeds: prevalence of hemivertebrae in French Bulldogs (Schlensker and Distl 2016), myxomatous mitral valve disease in Cavalier King Charles Spaniel (O’Brien et al. 2021), hip dysplasia in Labrador Retrievers (Kieler et al. 2024), malignancy risk of tumours in Pit Bull and Boxers (Pinello et al. 2022) and back problems in Dachshunds (Mogensen et al. 2011).

Welfare problems that stem from morphological selection, concern not only metabolic deficiencies as above mentioned, but also behavioural aspects. It is mainly anxiety and anxiety-like traits (such as noise sensitivity/reactivity, impulsivity, or (firework) fearfulness) that are suggested to be linked to some breeds selection [in Finnish pet dogs (Salonen et al. 2020), in Norwegian standard poodles (Handegard et al. 2023)]. In addition, repetitive behaviour [in Finnish pet dogs (Sulkama et al. 2022) or aggressive behaviour, in American and Belgian Malinois (Lit et al. 2013) and in North American Border Collies (Van Buren et al. 2021)] have been linked to genetic mutations.

In addition to these temporarily fashionable breeds that suffer from high inbreeding pressure, differential breeding strategies are also implemented to produce individuals with phenotypic traits considered desirable by owners but detrimental to the animals (Lampi et al. 2020). Although these animals are still regarded as representatives of their breed, this extreme breeding has been criticized for several years. For example, professional associations such as the Federation of Veterinarians of Europe (FVE, fve.org/publications/breeding-for-extreme-conformations-what-is-the-problem/) and the Nordic Kennel Union (Breed Specific Instructions, skk.se/en/NKU-home/projects/breed-specific-instructions/) have voiced concerns. The most well-known example of extreme breeding is the brachycephalic breeds, many of whose members suffer from Brachycephalic Obstructive Airway Syndrome (BOAS). The extreme shortening of the nose in these dogs is associated with respiratory difficulties, gastrointestinal disorders, hypertension, poor thermoregulation, and reduced exercise tolerance (Mitze et al. 2022). The presence of health issues “related to breed” among an increasing number of its members leads breeders, owners, and health specialists such as veterinarians to regard them as “normal” (Packer and Tivers 2015). However, this represents a genuine welfare issue that even treatments and other surgical interventions cannot always rectify.

Finally, in addition to the heightened selection for fashionable physical traits within a single breed, Europe is witnessing a phenomenon that emerged in North America nearly 35 years ago: the creation of hybrid dogs from two purebred parents. These animals are called designer dogs (Hladky-Krage and Hoffman 2022). Although they represent mixed-breed dogs, they are to be distinguished from true mixed-breed dogs, which result from uncontrolled canine crossbreeding, sometimes involving more than two breeds in varying proportions within the parental genetics. These designer dogs are the random result of parental genetic mixing, resulting in an entirely unpredictable phenotype.

## DYSFUNCTIONAL HUMAN PSYCHOLOGICAL PROCESSES

Research on human-animal relationships has garnered much attention in recent decades [for a history of human-animal interactions (HAI); see Fine et al. (2019)]. From the animal’s perspective, its social and cognitive abilities offer insight into its presence alongside humans. Animals can discriminate between different humans, perceive their emotions, attribute intentions to them, communicate and learn from them (Jardat and Lansade 2022). Conversely, aside from zoonoses and allergies (Judson and Rabinowitz 2021; Schoos et al. 2021), studies on interactions with animals have shown both positive and negative effects on humans, highlighting the complexity of this relationship and its dependence on numerous factors. For instance, an animal can positively influence human health by encouraging more musculoskeletal and cardiovascular activities [e.g., to varying degrees in elderly individuals (Gee and Mueller 2019) and in adolescents (Pajaujiene and Petrigna 2024)]. However, the responsibilities associated with animal care, including the provision of food, healthcare and housing, can give rise to concerns in the event of illness or behavioural issues (Barcelos et al. 2023).

The interpretation of the mental health of humans surrounded with animals is, however, more debated. On one hand, mental health is seen to be improved by animals’ presence. A systematic review of the literature shows that animals, either as privately-owned pets or in animal-assisted interventions, have a positive impact on the quality of life and mental health of people over 60 years old (Hughes et al. 2020). The meta-analysis by Martins et al. (2023) regarding pet ownership presents a positive but weak impact on human mental health. However, animal ownership could also be an indicator of a mental health problem. For instance, Ellis et al. (2024) studied various concepts from human relational science, applying them to relationships with pets (dogs and cats) and comparing them to various human mental outcomes. Although the interpretation of these concepts is unidirectional in Ellis et al.’s work, as it is based solely on the owner’s responses to a questionnaire about their pet, these authors highlighted some predictors (positive or negative) of depression, anxiety, affect, and feelings of loneliness that are: the respondent’s attachment to their pet, his/her perceived pet responsiveness (e.g., “*my pet knows me well*”), his/her perceived pet insensitivity (e.g., “*my pet is not attentive to my needs*”), and the owner’s self-expansion [that is the fundamental drive to enhance one’s self-concept through new experiences and close relationships (Aron et al. 2022)]. Similarly, the systematic review by McGrath et al. (2024) shows a positive association between the presence of a cat in people’s lives (in a broad sense: ownership, contact, or bite) and an increased risk of being diagnosed with schizophrenia-related outcomes (i.e., schizophrenia, schizoaffective disorder, and psychosis including bipolar disorder).

Several characteristics that govern human relationships can be observed between humans and other animals. Empathy, attachment, and anthropomorphism are considered essential for a harmonious relationship with animals and for ensuring their welfare (Prato-Previde et al. 2022). If these characteristics are insufficient, it may result in negligence, abuse, and/or cruelty towards animals, depending on the severity of the impact (Alleyne and Parfitt 2019). Conversely, if exaggerated, these characteristics can also be detrimental to animal welfare. For instance, animal rescuers suffering from hoarding disorder, known as Noah’s syndrome, attempt to rescue abandoned animals but end up taking in an excessive number, sometimes of different species, to the detriment of the Animals’ Five Freedoms: issues with food/water provision, confinement, poor health, injuries, and stress (Arluke et al. 2017).

Although many unknowns remain, some factors may explain the attraction of a significant number of humans to caring for animals with deleterious phenotypes, sometimes very morbid or even fatal. One might think that a lack of knowledge is one of the causes of this phenomenon, despite the statements of prospective owners regarding the information they gather from various sources before choosing a dog (Mead et al. 2024). However, it is paradoxical to note, regarding brachycephalic dog breeds, that the poor quality of life of these dogs and the associated health problems are not sufficient factors to consider their well-being compromised (Packer et al. 2019). These authors suggest that a certain form of cognitive dissonance might be at play, explaining the misperceptions of these owners. They believe that their animal, although in poorer health, is not less healthy than the average of other individuals of its breed.

The author suggests that the owner’s self-expansion mentioned above could be another explanation for the interest in these deleterious canine phenotypes, at least among certain owners. To what extent pet parenting [i.e., the choice to care for pets rather than raising children, as described in the US (Volsche 2021) and in China (Guo et al. 2021)] fails to meet the need for self-expansion remains to be explored. It is also possible that these animals are regarded as objects or possessions that the owner may use and abuse, justifying the lack of consideration for their well-being (Alleyne and Parfitt 2019). Finally, these animals could be seen as selfobjects in the sense described by (Brown 2007), allowing the owner to satisfy their self-esteem, ensure their self-cohesion, and provide them with calm, solace, and acceptance. These various hypotheses remain to be explored in order to gain a better understanding of extreme breeding and, consequently, to propose suggestions aimed at reducing this animal suffering.

## SUGGESTIONS TO IMPROVE ANIMAL WELFARE

It must be acknowledged that dogs bear a heavy burden at the hands of human-controlled selection. The domestication process is no longer a win-win concept between these two species, as the author previously presented. Animal welfare, as a selection criterion, should guide canine breeding strategies by all involved stakeholders.

Indeed, pushing phenotypic criteria beyond limits (extreme breeding), restricting genetic exchanges between subpopulations of the same breed (inbreeding, impoverishment of genetic diversity), or creating “new temporary breeds” (e.g., Labradoodle, a cross between a purebred Labrador Retriever and Poodle) are becoming commonplace human actions, without sufficient ethical consideration. Thus, owners of dogs suffering from BOAS do not consider their animal to be at higher health risk than others. The care and attention it requires are seen as part of the constraints specific to its breed (Mitze et al. 2022). And the breed crosses are creating welfare problems as the expected Darwinian positive hybrid vigour is not observed when crossing purebred Labrador Retrievers and Poodles, In the Labradoodles studied by Oliver and Gould (2012), it was established by Nicholas et al. (2016) that the multifocal retinal dysplasia had a higher prevalence than that observed in their parental breeds. In addition, due to a lack of information about the crosses made to obtain these Labradoodles (were they all F1 products?), the hybrid vigour cannot be estimated.

Various proposals to improve the welfare of dogs have recently been formulated. It would be interesting for researchers to focus on their implementation and evaluate their effectiveness in the near future. It has been suggested that dog owners be informed about the health problems associated with canine breeds by increasing dialogue with veterinarians in clinics (Janke et al. 2021) and also before purchasing the dog (Philpotts et al. 2024). For example, but this is far from the only one, informing owners about the longevity of animals depending on their breed (McMillan et al. 2024). For (future) owners seeking information on the internet and social media, the quality of the sources consulted must be indicated because responsible breeders (most of those affiliated with a canine federation), professional breeders who have made it their (sole) source of income, and veterinarians do not have the same interests in breeding (Kuhl et al. 2022).

The prevention of breeding deviations could be anticipated by understanding the mechanisms of genetic diversity fragmentation occurring within canine breeds [selection pressure on certain traits deemed desirable, in certain lineages or regions of the world (Kuhl et al. 2022)]. The correction of breeding deviations could also be evaluated by studying the underlying genomic mechanisms, starting with the current individuals and making the genomic status at the starting point known (Grall et al. 2012). Significant genetic modifications should not take long to observe. Indeed, Trut et al. (2009), in their domestication experiment with silver foxes, observed tail wagging, characteristic of dogs, after four generations. Two generations later, other typically canine behaviours were observed (whining, whimpering, and licking).

Canine federations worldwide play a crucial role in addressing the issue at hand. Wang et al. (2017) demonstrated that merging national pedigree databases from European countries (France, Sweden, and England), which provided access to unrelated sires and dams within these databases, helped to reduce or stabilise inbreeding over two generations in three out of the four breeds studied (Bullmastiff, English Setter, Bernese Mountain Dog, but not for the Labrador Retriever in France). They also found that the volume of gene exchange between countries was higher, increasing genetic variability within these breeds. However, this outcrossing effort needs to be sustained (over several years and involving a large number of animals) to have a long-term impact at the national population level, as demonstrated by Windig and Doekes (2018) through computer simulations. In a similar vein, Leroy and Rognon (2012) simulated the effect of various mating restrictions in four French dog breeds of different population sizes (Braque Saint Germain, Berger des Pyrénées, Coton de Tulear, Epagneul Breton) to figure out how to control for one genetic defect. Mating restrictions were applied for one year and included one of the following measures: removing diseased animals from breeding, removing animals carrying the genetic defect, allowing breeding with heterozygote animals but subsequently removing their offspring carrying the defect, and to control the sire effect, limiting the number of litters per male. Results of these simulations showed a favourable effect of these measures on the transmission of genetic defects, though to varying degrees depending on the breed’s population size, the severity of the genetic defect, and its initial frequency. A case-by-case analysis should be considered when applying these measures in practice.

Finally, veterinarians must be made aware of these abuses in dog breeding. Their education, webinars, workshops, and seminars can help in that sense [for example, some vets still consider BOAS-associated problems as “typical of the breeds” but brachycephalic breeds have been shown to be up to 5 times more likely than others to present malformed puppies (Estevam et al. 2022)]. Also thorough education of veterinary students at universities needs to be stressed. Veterinarians will then be fully informed and competent to advise and help dog breeders and their future owners in their choices (Czerwinski et al. 2016). For example, veterinarians could offer their clients the FVE’s leaflet: Breeding for extreme conformations: what is the problem? (https://fve.org/publications/breeding-for-extreme-conformations-what-is-the-problem/), organise (free) pre-purchase consultations on wise dog selection (Mead et al. 2024) in order to reduce the demand of dogs that suffer from morphological deviations, metabolic or behavioural defects, negatively influencing their welfare (Sandoe et al. 2017).

To be effective, all these suggestions for improving canine welfare must be assessed against real-world conditions. To avoid resistance and promote compliance with any forthcoming measures – whether they originate from canine federations, international or national animal welfare legislation – it is essential that policymakers are aware of their target audience [since perceptions and experiences of owners, breeders and veterinarians are variable (Asbjer et al. 2024)], and of the psychological mechanisms underlying all changes (Ophorst et al. 2023).

## CONCLUSION

Originally, selective breeding aimed to produce animals carrying traits of human interest. It is on the basis of this principle that canine breeds were developed. Over time, genetic diversity has diminished and the spread of hereditary defects within these breeds has increased. Breeding strategies that prioritise animal welfare must be implemented promptly to ensure a satisfactory quality of life for the animals, enable breeders to continue their activities, and allow owners to establish a fulfilling relationship with their pets.
